# Hyperbaric oxygen therapy for Crohn’s disease complications: What do we know? –Authors’ reply

**DOI:** 10.1177/2050640620945090

**Published:** 2020-07-19

**Authors:** CA Lansdorp, RA van Hulst

**Affiliations:** Amsterdam UMC, location AMC, Department of Anaesthesiology/Hyperbaric Medicine, Amsterdam, the Netherlands

We want to thank Parra et al. for their interest in our article “Wound healing of metastatic perineal Crohn’s disease using hyperbaric oxygen therapy: A case series”, and for sharing their experience with the treatment of patients with therapy-refractory Crohn’s disease (CD) complications.^1,2^

In their letter, the authors explain that the use of adjunctive hyperbaric oxygen (HBO) therapy is not a novel therapeutic strategy and that it has been used in therapeutic algorithms for more than a decade. Indeed, the use of HBO in inflammatory bowel disease (IBD) has been reported as early as 1989, and an emerging amount of scientific evidence is available.^3,4^ For biopsy-proven metastatic CD, however, the use of HBO had only been reported once before.^3^

Although there is an increase in published studies about the therapy and its effects, HBO is not (yet) an established indication for IBD, neither in Europe nor in the USA.^5,6^ In current practice, its application mostly remains at the discretion of the treating physician and the availability of hyperbaric chambers. One of the underlying reasons for this is the quality of the current evidence: a substantial proportion of studies that have been published have a small sample size and a high risk of bias.^4^ The authors state in their letter that it would be ethically questionable to add a control group to a study because patients with an aggressive condition and limited therapeutic options would be denied a possibility of treatment with HBO. Performing (sham) controlled trials using HBO can indeed be challenging (though not impossible), but high-quality research is necessary precisely to make sure that the treatment is available for those patients that might benefit from it.^7^

We would like to thank the authors for their suggestion of performing a subsequent study on metastatic CD and HBO; a multicentre or international approach could indeed result in the required sample size. The HOT-TOPIC trial that the authors refer to has been executed as a first step in expanding the knowledge about HBO for perianal fistulas in CD; its short-term outcomes will be submitted for publication in the near future.^8^
